# The Use of Middle Meningeal Artery Embolization to Treat Chronic Subdural Hematoma in the Pediatric Population: A Review of the Literature

**DOI:** 10.7759/cureus.61874

**Published:** 2024-06-07

**Authors:** Abdulrahman D Alofi, Thamer H Alsharif, Abdulrahman A Alshamrani, Adel A Alsulami, Zeyad Alamri, Mashhour A Alsuwat

**Affiliations:** 1 Neurosurgery, King Abdulaziz Specialist Hospital, Taif, Taif, SAU; 2 Neurosurgery, Royal College of Surgeons in Ireland, Dublin, IRL; 3 Neurosurgery, Al Noor Hospital, Jeddah, SAU; 4 Neurosurgery, King Abdulaziz University, Jeddah, SAU; 5 Neurosurgery, King Faisal Specialist Hospital and Research Centre, Jeddah, SAU

**Keywords:** pediatric subdural hematoma, middle meningeal artery embolization, pediatric head injury, chronic subdural hematoma (csdh), pediatric head trauma

## Abstract

Chronic subdural hematoma (cSDH) is rare in the pediatric population and typically arises from various causes. These include trauma (accidental, non-accidental, or birth-related injuries), coagulopathies (such as hemophilia or von Willebrand disease), vascular malformations (such as arteriovenous malformations), and complications from previous surgeries. These diverse etiologies contribute to the complexity of managing this condition. Although middle meningeal artery (MMA) embolization is proven effective in adults, limited studies have investigated its applicability in pediatrics. This study aims to assess the efficacy, safety, and outcomes of MMA embolization in the pediatric age group, guiding future research and treatment strategies. A systematic review of the literature was conducted using PubMed, Web of Science, and Embase. No restrictions were applied regarding publication status or follow-up duration. The inclusion criteria were studies that integrated MMA embolization as a treatment for cSDH in pediatric patients. Data extracted included patient sample and characteristics, cSDH etiology and characteristics, prior intervention, procedural technique and indication, and clinical and radiological outcomes. Twelve studies were included in the review, comprising a total of 14 patients. There were no randomized clinical trials or large-scale cohort studies. The included literature consisted of 11 case reports and one case series, and the results described a clinical and radiological outcome in a varied mix of patients with different characteristics and backgrounds for cSDH. No neurological complications attributed to MMA embolization were reported. Follow-up showed resolved or decreased size of cSDH in all patients except for one, who experienced hematoma expansion despite treatment. MMA embolization may be considered a primary or adjuvant treatment modality for cSDH in the pediatric population. However, further research is needed to investigate the impact of different etiologies on outcomes and to highlight long-term complications and results.

## Introduction and background

Chronic subdural hematoma (cSDH) is a commonly observed pathology in the elderly, predominantly attributed to age-related brain atrophy [1]. Conversely, its occurrence in pediatric age groups is relatively infrequent and has diverse underlying causes. These may include head injuries, whether accidental or non-accidental, as well as coagulopathies and vascular malformations [2-5].

There are different options for the management of cSDH in the pediatric population, ranging from watchful waiting with serial image monitoring for asymptomatic small lesions to subdural puncture in patients with open fontanelle, burr hole drainage, placement of a subdural shunt, and craniotomy for evacuation [6,7]. However, some of these patients are unfit for invasive procedures or need to remain on anticoagulation (AC) therapy, which adds to the complexity of surgical management and increases the risks of procedural complications and recurrence.

Studies have consistently shown that middle meningeal artery (MMA) embolization is a feasible option and is associated with lower rates of hematoma recurrence and surgical rescue compared to conventional treatment in adults [8-10]. This minimally invasive approach may reduce the need for surgical intervention and is a viable option for the treatment of cSDH in the pediatric population, whose condition is suboptimal or who are medically unfit for surgery.

The aim of this study is to perform a systematic review of published studies to evaluate the efficacy, safety, and outcomes of MMA embolization in pediatric patients, thereby helping to identify the gaps in our knowledge, highlight any possible inconsistencies, and guide treatment planning and future research.

## Review

Materials and methods

Search Strategy

The systematic review was conducted according to the Preferred Reporting Items for Systematic reviews and Meta-Analyses (PRISMA) guidelines. The search for related studies was done by two independent researchers using PubMed, Web of Science, and Embase. The search was from inception until March 10, 2024. The review is designed to assess the efficacy, safety, and outcomes of using MMA embolization in the pediatric population for the treatment of cSDH. There was no restriction on publication status or follow-up. The primary search terms used in the search were as follows: (subdural hematoma OR intracranial subdural hematoma OR chronic subdural hematoma) AND (pediatric OR children) AND (embolization).

Study Selection

All studies identified by the database search were exported to Excel by two independent researchers, and duplicates were excluded. Titles and abstracts of all the search results were reviewed for inclusion and exclusion criteria. Studies were excluded if (1) there were no complete texts, including abstracts, editorial letters, comments, etc.; (2) there were studies without follow-up; and (3) there were studies that did not involve cSDH. The inclusion criteria were the following: (1) participants in the study were humans; (2) the study was published in English; (3) subjects were under the age of 18; and (4) the study included at least one case to validate the use of MMA embolization for cSDH treatment.

Data Extraction

From each included study, the following data were extracted independently by the two reviewers: publication year and author, study design and patient number, patient sample and characteristics (including age, gender, presenting symptoms, comorbidities, and diagnosis), etiology and characteristics of cSDH, whether there was any prior intervention for the same lesion, details and indication of the procedure studied, the reported outcomes, and any available follow-up.

Risk of Bias Assessment

All the articles included in this review were case reports, which inherently have a high potential for reporting and publication bias. The Risk of Bias In Non-randomized Studies of Interventions (ROBINS-I) tool was used to evaluate the included studies and determine their overall risk of bias.

Results

Study Selection

**Figure 1:**
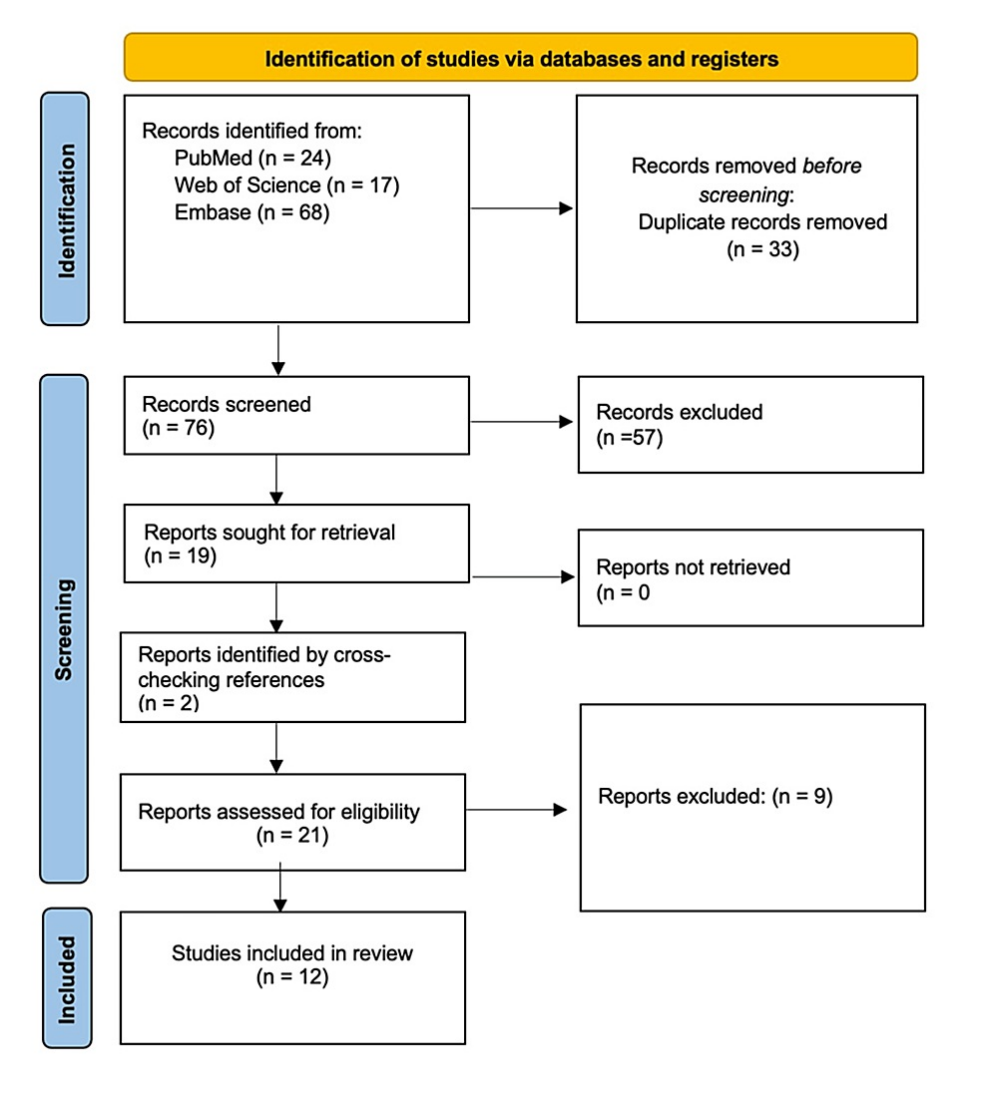
PRISMA flowchart PRISMA, Preferred Reporting Items for Systematic reviews and Meta-Analyses

As shown in Figure 1, the conducted search resulted in 109 records: 24 in PubMed, 17 in Web of Science, and 68 in Embase. A total of 33 duplicate studies were excluded. After title and abstract screening, 19 studies were included for full-text review for eligibility. A total of 10 articles met all inclusion and exclusion criteria and were included in this review. Additional two studies were identified by reviewing reference sections from the included studies. Finally, a total of 12 studies were included in the final systematic review.

Studies Characteristics

As shown in Table 1, the included studies were published between 2015 and 2023, with 10 of them being between 2021 and 2023. One study is a case series consisting of three patients. The remaining 11 studies were case reports, yielding a total of 14 patients.

**Table 1:** Study characteristics and review of the literature AC, anticoagulation; LcSDH, chronic subdural hematoma; MMA, middle meningeal artery; n-BCA, n-butyl cyanoacrylate; SDH, subdural hematoma

Author, year, and study design	Age and gender	Presentation	Etiology	Prior intervention	Procedural indication	Procedure	Clinical outcome	Radiological outcome	Follow-up
Kang et al. (2015) [11], Case report	13-year-old male	Headache, nausea, and vomiting	Arachnoid cyst	Burr hole drainage twice for the right cSDH	Prevention of recurrent right cSDH	Right MMA embolization with coils	Clinically intact	Resolution and no recurrence at 3-month follow-up CT	5 years
Higaki et al. (2017) [12], Case report	7-year-old male	Asymptomatic	Hemophilia A	Burr hole drainage followed by craniotomy for left acute subdural hematoma evacuation	Prevention of recurrent refractory left cSDH	Left MMA embolization with particles	None reported	Complete resolution of the SDH on follow-up CT	9 months
Shigematsu et al. (2021) [13], Case report	5-month-old female	Asymptomatic history of left middle cerebral artery stroke ventricular assisted device on antiplatelet and AC	AC therapy	None	Primary treatment for expanding bilateral cSDH	Bilateral MMA embolization with n-BCA	Neurologically free	Decreased hematoma size on follow-up CT at 5 months	5 months
Faber et al. (2021) [14], Case report	18-month-old male	Enlarged head circumference, intermittent dysconjugate gaze, and a medially deviated left eye	Non-accidental trauma	Burr hole drainage of bilateral subdural hematoma followed by craniotomy for right subdural hematoma evacuation	Prevention of recurrent right cSDH	Right MMA embolization with onyx	Meeting developmental milestones with a near-resolution dysconjugate gaze	Near total resolution of the right cSDH on CT at 6 months	8 months
Yazawa et al. (2022) [15], Case report	2-year-old female	Vomiting, ventricular-assisted device on antiplatelet and AC	AC therapy	Craniotomy for right acute on top of cSDH, followed by craniotomy for left acute on top of cSDH	Prevention of the recurrence of left cSDH	Left MMA embolization with n-BCA; *n-BCA migrated into intracranial vessels via undetected transdural anastomosis	No neurological symptoms	Decreased hematoma size on follow-up CT at 2 months	8 months; *died of ventricular-assisted device infection at 8 months
Ravi et al. (2022) [16], Case report	2-year-old female	Third nerve palsy	Non-accidental trauma	Decompressive craniectomy for right acute subdural hematoma evacuation	To treat reaccumulating right cSDH after craniectomy	Right MMA embolization with onyx	Stable third nerve palsy, general improvement of neurological condition	Near total resolution of cSDH at 6-month follow-up CT	6 months
Souter et al. (2022) [17], Case report	15-month-old male	The neurologically intact ventricular-assisted device on antiplatelet and AC	AC therapy	None; medically unfit for surgery	Primary treatment of an expanding acute on top of a chronic bilateral subdural hematoma	Bilateral MMA embolization with embospheres and coils	Neurologically intact	Stable left cSDH and expanding recurrent right cSDH	3 months
Paro et al. (2023) [18], Case report	8-year-old male	Headache; medically free	Unknown	None	Stable patient with right acute on top of cSDH undergoing digital subtraction angiography	Right MMA embolization with microparticles and coil	Clinically intact	Complete resolution on MRI at 6 months	6 months
Gal et al. (2023) [19], Case report	15-month-old female	Irritability, apneic episodes, seizures, and global developmental delay	Non-accidental trauma	Bilateral burr hole drainage twice for bilateral cSDH	Prevention of the recurrence of bilateral cSDH	Bilateral MMA embolization with a liquid embolic agent	Developmental delay, seizure free	Decrease in the size of the subdural collection at the 10-month follow-up MRI	10 months
Vazquez et al. (2024) [20], Case report	13-year-old male	Headache and vomiting	Craniotomy for tumor resection and ventriculoperitoneal shunt insertion	Bilateral craniotomy for bilateral subdural hematoma	To reduce the risk of enlargement or recurrence of persistent subdural hematoma	Bilateral MMA embolization with particles and coils	Occasional headache	Near total resolution of bilateral SDH at the 9-month follow-up MRI	9 months
Marnat [21] 2023 Case report	16-year-old male	Asymptomatic	Ruptured left arachnoid cyst	Burr hole drainage and subduroperitoneal shunt for left cSDH	To treat reaccumulating left cSDH	Left MMA embolization by microparticles	Asymptomatic	Complete resolution at 6 months on follow-up MRI	6 months
Coyle et al. (2023) [22], Case series	8-year-old female	Headache post-Fontan procedure and stenting on AC	AC therapy	Burr hole drainage complicated by a left acute subdural hematoma	To treat reaccumulating left cSDH	Left MMA embolization by particles	None reported	Complete resolution at 3 months on follow-up CT	24 months
	8-month-old female	Lethargy and seizures; multiple thrombotic events in chronic AC	AC therapy	Craniotomy and evacuation of the right acute on top of cSDH	Prevention of reaccumulation of the right subdural hematoma	Right MMA embolization with particles	None reported	No reaccumulation during follow-up	17 months
	3-year-old male	Intact low-grade glioma and obstructive hydrocephalus treated by endoscopic third ventriculostomy	Post-craniotomy for tumor biopsy	None	Primary treatment of left cSDH	Left MMA embolization with onyx	None reported	Near total resolution of the left cSDH at 1-month follow-up MRI	12 months

Sample Characteristics

As shown, patients’ ages ranged from seven months to 16 years old, with a median of three years old (IQR 1.25-13). Five patients were on AC therapy, three of whom had cardiac myopathies and were on ventricular-assisted devices waiting for a heart transplant, one patient had a post-Fontan procedure and stenting, and one had multiple thrombotic events. Three patients had a history of non-accidental head trauma (NAT). Two patients had brain tumors. Two patients had arachnoid cysts that were ruptured in one of them. One patient was diagnosed with hemophilia A, and one patient was medically free with no obvious etiology for the cSDH. Four of the patients had bilateral cSDH and underwent bilateral MMA embolization. Four patients had no prior intervention. Ten patients had prior surgical interventions with a total of 14 surgeries: unilateral burr hole drainage (n = 5), bilateral burr hole drainage (n = 3), unilateral craniotomy (n = 4), bilateral craniotomy (n = 1), and subdural-peritoneal shunt placement (n = 1).

Intervention Characteristics

MMA embolization was the primary treatment for four out of 14 patients. Embolization was done bilaterally in fur patients as well, while the remaining 10 had unilateral embolization. Different embolization materials were used in the studies: particles (n = 4), coils (n = 4), onyx (n = 3), microparticles (n = 2), n-BCA (n = 2), embospheres (n = 1), and liquid embolic agents (n = 1). Procedural complications were only reported in one case with n-BCA embolic material migrating into the intracranial vessel through undetected transdural anastomosis; however, the patient had no clinical manifestation.

Outcome

The follow-up period ranged from three months to five years, with a median of 8.5 months (IQR 6-12). Six patients had complete resolution of the cSDH on follow-up images; four had near total resolution; three were reported to have decreased the size of the hematoma; and one patient who underwent bilateral MMA embolization had stable size on one side and an expanding hematoma on the other. One patient reported having occasional headaches during follow-ups. One patient died of a ventricular assisting device infection eight months after MMA embolization. One patient who is a victim of NAT was reported to have a developmental delay that was attributed to NAT rather than the MMA embolization. All the remaining 11 patients were either neurologically free or had improved their previously existing symptoms, with no report of newly developed neurological deficits.

Discussion

cSDH is a common pathology in the adult population; however, it is also seen in pediatric patients. The presence of cSDH in pediatrics has been associated with multiple factors in the literature, including NAT, AC therapy, CSF over-drainage, arachnoid cyst rupture, and postoperatively following resection of brain tumors. The complexity of the existent comorbidities in those patients presents MMA embolization as an appealing, less invasive option of treatment for cSDH.

MMA embolization is a well-established modality of treatment for cSDH in adult patients. A meta-analysis of 1,416 patients showed a lower rate of recurrence and surgical rescue, with a comparable rate of complications compared to conventional management (surgical and conservative approaches) [10]. Despite that, little is known about the safety and applicability of this approach in pediatrics due to limited publications.

Different embolic agents were used among the included studies, with particles, coils, and onyx being the most common. There was no specific indication for the use of one embolic agent over the others. In one study, n-BCA migrated into intracranial vessels via undetected transdural anastomosis, but the patient remained neurologically intact [15]. Although the youngest patient in the reviewed literature was seven months old, there were no reported procedural or vascular access complications. Despite that, the applications of MMA embolization in infants present distinct challenges as they have smaller caliber vessels, raising the difficulty of securing vascular access and the potential risk of vascular dissection.

Out of 14 patients included in this study, there was one reported mortality due to ventricular assisting device infection; additionally, one patient had a recurrence and expansion of cSDH after embolization [17]. The remaining patients had complete or near-complete resolution of the hematoma with no recurrence. These are promising outcomes that support the safety and applicability of MME embolization in pediatric patients. However, the long-term effect of devascularization of the dura matter and its effect on skull development in pediatrics warrants longer follow-up and further investigation. Moreover, endovascular intervention carries the risks of stroke, visual loss, contrast, and radiation exposure. These risks must be addressed and accounted for. Pre-procedural angiography to study collateral circulation and the involvement of a neurointerventionalist with experience in pediatrics might help to minimize the risks of any procedural complication.

Indications for MMA embolization in the reviewed literature were either to primarily treat cSDH or as an adjuvant treatment for treating recurrence or prevention of reaccumulation of the hematoma. Patient selection was mainly due to the presence of other comorbidities or the required use of AC therapy, putting those patients at high risk for any surgical evacuation. Taking into consideration that the standard operative approach has a 30% rate of recurrence [23], MMA embolization might be an effective choice, being a less invasive modality with the ability to eliminate blood supply that contributes to hematoma formation and expansion. However, it is limited to hematomas without significant mass effects, as it lacks the ability to immediately lower high intracranial pressure and relieve mass effects that are only achievable by surgery [24].

The limitations of this study encompass several aspects. Firstly, there are inherent risks of bias in case reports, compounded by publication bias, which may skew the reported outcomes toward positive results. Additionally, the wide variability in the duration of follow-up periods across studies presents challenges in drawing definitive conclusions regarding recurrence rates and long-term neurological outcomes. Follow-up periods ranged from three months to five years, posing difficulties in assessing the sustainability of treatment effects over time.

Moreover, significant heterogeneity exists in the studied sample, procedural techniques, and embolic agents employed. This diversity complicates the interpretation and generalizability of the findings. Considering these factors collectively, applying the results in real clinical practice with a high degree of confidence becomes challenging. Nevertheless, despite these limitations, the findings of this study serve as a proof of concept for the potential application of MMA embolization in the pediatric population.

## Conclusions

MMA embolization might be considered a primary or adjuvant treatment modality for cSDH in the pediatric population. The reviewed literature showed that it is effective and could be safely applied. However, further research is needed to study the effect of different etiologies on the outcome and to highlight long-term complications and results.
